# 

**Published:** 1868-12-15

**Authors:** 


### *CONTENTS:*

ORIGINAL PAPERS:

Case of Hepatic Abscess—Evacuation of Pus via Bronchial Tubes—Recovery By S. T. Odell, St. Louis, Miami Co., Kansas ..... 780
Novel Treatment of Sunstroke ..... 784
Dilation of the Os Uteri and Perinæum during Labor. By George Kilner, M.D., of Sullivan, Ill ..... 785
Digitalis—Its Use in Suppression of Urine. By James T. Newman, M.D ..... 790
Interesting Cases Successfully Treated with Pepsine ..... 792
Ovariotomy ..... 794

CORRESPONDENCE ..... 795

BOOK NOTICES ..... 796

EDITORIAL:

Abortion ..... 797
To Readers and Correspondents ..... 800
Literary Exchanges ..... 801
An Important Matter ..... 802
Introductory Addresses ..... 803
Book Notices, etc ..... 803

OBITUARY ..... 803
California Wines ..... 804
LOOT ..... 805
Foreign Items ..... 809

